# Understanding the Heat Shock Response in the Sea Cucumber *Apostichopus japonicus*, Using iTRAQ-Based Proteomics

**DOI:** 10.3390/ijms17020150

**Published:** 2016-02-04

**Authors:** Dongxue Xu, Lina Sun, Shilin Liu, Libin Zhang, Hongsheng Yang

**Affiliations:** 1 Key Laboratory of Marine Ecology and Environmental Sciences, Institute of Oceanology, Chinese Academy of Sciences, Qingdao 266071, China; dongxuexu1989@126.com (D.X.); shlliu72@126.com (S.L.); zhanglibin@qdio.ac.cn (L.Z.); 2 University of Chinese Academy of Sciences, Beijing 100049, China

**Keywords:** *Apostichopus japonicus*, heat stress, iTRAQ, proteomics analysis

## Abstract

The sea cucumber *Apostichopus japonicus* is exploited as a commercial species owing to their high nutritive and medicinal value. Recent high summer temperatures have caused high mortality rates in *A. japonicus*. In this study, we applied the isobaric tag for relative and absolute quantitation (iTRAQ) technique to investigate the global protein expression profile under an acute short-term (48 h) heat stress. In total, 3432 proteins were identified, and 127 proteins showed significant heat stress responses, with 61 upregulated proteins and 66 downregulated proteins. Our results suggest that heat stress influenced the expression of proteins involved in various biological processes, such as tissue protection and detoxification, lipid and amino acid metabolism, energy production and usage, transcription and translation, cell apoptosis, and cell proliferation. These findings provide a better understanding about the response and thermo-tolerance mechanisms of *A. japonicus* under heat stress.

## 1. Introduction

The effects of global warming include rising mean annual temperatures and dramatic increase in the frequency and amplitude of severe temperature events [1]. These fluctuations constitute a major threat to aquatic organisms, as they are naturally exposed to changing water temperature. The sea cucumber *Apostichopus japonicus*, is an echinoderm distributed along the coast of northern China, southeastern Russia, Japan, the Republic of Korea, and the Democratic People’s Republic of Korea [2]. *A. japonicus* has been exploited as a commercial species owing to their high nutritive and medicinal value. Temperature is the pivotal environmental factor affecting the growth and physiology of *A. japonicus* [3]. Recent high summer temperatures have caused high mortality rates in cultured *A. japonicus*. Therefore, a better understanding of the mechanisms involved in the *A. japonicus* heat shock response would be significant and would lay the theoretical foundation for breeding traits for thermo-tolerance. Though specific heat response genes, such as genes from the heat shock protein (HSP) family, have been characterized, a lack of transcriptome and proteome data severely hampers revealing global gene changes and the key pathways that are active in heat stressed *A. japonicus* [4,5,6].

Proteomic approaches have been used to identify stress-responsive genes and proteins regulated by high temperatures. Two-dimensional electrophoresis (2DE) is the most frequently utilized approach for a proteomic analysis. However, not all proteins are amenable to gels, and proteins in low abundance are hard to be characterized in 2DE approach [7]. Besides, the quantification accuracy and ability of 2DE to identify proteins may be compromised by co-migration or partial co-migration of proteins [8]. A new technique called iTRAQ (isobaric tag for relative and absolute quantitation) has become popular in proteomic analysis in recent years, which provides more reliable quantitative measurements and comparisons among samples [9]. Additionally, the iTRAQ approach has largely improved proteomic analyses throughput and has been used in pathway studies.

In the current study, we applied the iTRAQ technique to assess the proteomic changes in *A. japonicus* intestinal tissues after heat shock. Our results suggest that heat stress influenced the expression of proteins involved in diverse biological processes, such as tissue protection and detoxification, lipid and amino acid metabolism, energy production and usage, transcription and translation, cell apoptosis, and cell proliferation. These findings provide a better understanding of the response and thermo-tolerance mechanisms in *A. japonicus* under heat stress.

## 2. Results

### 2.1. Overview of the Proteomics Data

**Figure 1:**
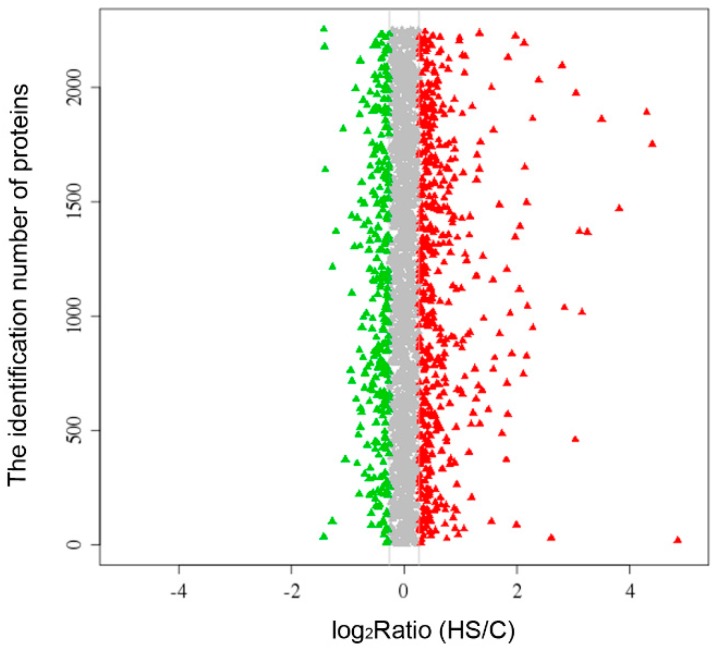
The change level of global proteins in the intestine in the heat shock group (HS) compared with the control group (C). Only the proteins with log_2_Ratio (HS/C) >0.26 or <−0.26 were colored (fold changes >1.20 as red and <0.83 as green).

The proteomics data have been deposited to the ProteomeXchange via the PRIDE (Database ID: PXD002660) [10]. Totally 272,754 spectra were obtained, of which 38,588 unique spectra were detected (Table 1). A total 3423 proteins were identified at a global false discovery rate of 1% (Table S1). The global expression changes of these proteins under heat stress were shown in Figure 1. Finally, 127 proteins showed significant heat stress responses, with 61 upregulated proteins (Table 2) and 66 downregulated proteins (Table 3).

**Table 1:** Overview of the proteomics sequencing results.

Group Name	Number
Total spectra	272,754
Spectra	41,330
Unique spectra	38,588
Peptide	10,908
Unique peptide	10,486
Protein	3432
Upregulated protein	61
Downregulated protein	66

**Table 2:** Sixty-one upregulated proteins under heat stress in the intestine of the sea cucumber *A. japonicas*.

Accession Number	Protein Description	HS *vs.* Control	
		Mean	SD
**HSPs and Related Proteins**			
Unigene28963	heat shock protein 90	6.10	4.25
CL6821.Contig1	heat shock protein 70	1.56	0.62
CL5625.Contig2	heat shock protein 110	1.41	0.22
Unigene15437	heat shock protein 10	1.20	0.01
CL12434.Contig1	heat repeat-containing protein 7A	1.36	0.19
**Detoxification and Tissue Protection**			
Unigene61290	glutathione *S*-transferase	1.36	0.07
CL6008.Contig2	glutathione *S*-transferase α-4-like, partial	2.23	0.72
Unigene25399	sigma class glutathione *S*-transferase 2	1.29	0.26
CL7884.Contig2	phospholipid hydroperoxide glutathione peroxidase	1.45	0.16
Unigene29285	prostaglandin D2 synthase, hematopoietic-like	1.53	0.35
Unigene25766	cytochrome P450 4V2-like	2.43	1.45
**Cell Apoptosis and Proliferation**			
Unigene29013	apoptosis-inducing factor 1, mitochondrial-like, partial	1.28	0.20
CL4411.Contig2	prohibitin-like	1.19	0.12
CL2387.Contig1	autocrine proliferation repressor protein A-like	3.57	3.08
Unigene33274	suppression of tumorigenicity 13 (colon carcinoma) (Hsp70 interacting protein)	1.27	0.09
CL10790.Contig2	erlin-1	1.29	0.04
Unigene35102	mesoderm-specific transcript protein (MEST)	2.61	2.19
**Lipid Transport and Metabolism**			
Unigene64084	long-chain specific acyl-CoA dehydrogenase	1.41	0.24
Unigene20467	long-chain specific acyl-CoA dehydrogenase, mitochondrial-like	2.22	0.72
Unigene22338	short chain dehydrogenase/reductase family 16C, member 5-like	1.74	0.58
Unigene4389	17-β-hydroxysteroid dehydrogenase type 4	1.94	1.11
Unigene5795	11-β-hydroxysteroid dehydrogenase	2.54	1.24
Unigene6420	enoyl-CoA Hydratase family member-like	1.62	0.33
CL12084.Contig1	enoyl-CoA hydratase, mitochondrial-like	1.30	0.16
CL759.Contig2	hydroxyacyl-Coenzyme A dehydrogenase	1.34	0.31
Unigene19362	epidermal retinol dehydrogenase 2-like	1.97	0.83
Unigene15259	carnitine *O*-palmitoyltransferase 2, mitochondrial-like	1.21	0.06
Unigene11008	non-specific lipid-transfer protein-like	2.99	1.98
CL4289.Contig1	nuclear progesterone receptor	1.46	0.23
CL6901.Contig2	2′-deoxynucleoside 5′-phosphate *N*-hydrolase 1	1.43	0.27
CL8136.Contig1	acyl-CoA-binding protein like, ACBP2	2.33	1.46
Unigene18754	oxysterol-binding protein-related protein 9	1.20	0.18
**Carbohydrate Transport and Metabolism**			
Unigene2131	lactase	1.48	0.10
**Amino Acid Transport and Metabolism**			
CL4095.Contig2	sphingosine-1-phosphate lyase 1	1.33	0.27
Unigene23212	branched-chain-amino-acid aminotransferase-like protein 1	1.39	0.32
**Energy Production and Conversion**			
CL10773.Contig1	isocitrate dehydrogenase	1.68	0.19
Unigene18857	electron transfer flavoprotein subunit α, mitochondrial-like	1.27	0.25
CL6007.Contig1	aldehyde dehydrogenase, dimeric NADP-preferring isoform	1.37	0.22
Unigene22955	α-methylacyl-CoA racemase-like	1.65	0.66
Unigene175	d-glucosyl-*N*-acylsphingosine glucohydrolase	1.85	0.31
Unigene22578	α-galactosidase	1.91	1.26
Unigene29260	ATPase family AAA domain-containing protein 1	1.35	0.05
CL5389.Contig1	ATPase inhibitor, mitochondrial-like	1.31	0.21
**Protein Synthesis**			
CL9215.Contig1	aspartyl-tRNA synthetase	1.17	0.11
CL7807.Contig3	RNA-binding motif protein, X chromosome	1.25	0.19
Unigene8195	elongation factor Tu, mitochondrial-like	1.34	0.20
**Others/Uncharacterized**			
Unigene49395	toposome	1.83	0.81
Unigene28479	natterin-3-like	2.63	1.01
CL6732.Contig2	calpain-5 isoform 2	1.38	0.37
Unigene22143	phospholipase C delta isoform	2.55	0.58
CL1115.Contig1	endophilin-B1-like isoform 1	1.32	0.19
Unigene322	myosin VIb-like	1.32	0.24
CL7807.Contig3	atlastin-2	1.33	0.23
CL9074.Contig2	cysteine rich protein 1	1.59	0.18
Unigene11767	suppressor of G2 allele of SKP1 homolog	1.53	0.14
CL8638.Contig1	development-specific protein LVN1.2	1.71	0.62
Unigene22386	uncharacterized	1.39	0.35
Unigene1947	uncharacterized	3.76	1.54
Unigene5634	uncharacterized	1.66	0.38
Unigene16247	uncharacterized	2.11	1.58
Unigene62712	uncharacterized	11.32	9.13

**Table 3:** Sixty-six downregulated proteins under heat stress in the intestine of the sea cucumber *A. japonicas*.

Accession Number	Protein Description	HS *vs.* Control	
		Mean	SD
**Cytoskeletal Proteins**			
Unigene32477	twitchin-like	0.67	0.21
Unigene32260	laminin subunit α-like	0.68	0.13
CL221.Contig4	α-actinin-like	0.76	0.10
Unigene3881	galectin-9-like	0.56	0.29
Unigene27394	fibrillin-1-like	0.52	0.17
CL5005.Contig4	cohesin subunit SA-1-like	0.69	0.25
Unigene9716	titin isoform 3	0.72	0.17
CL3832.Contig7	filamin-C isoform 1	0.83	0.01
Unigene30625	muscle M-line assembly protein unc-89-like	0.71	0.10
**Transcription and Translation**			
Unigene29879	60S ribosomal protein L8-like	0.79	0.11
Unigene26472	60S ribosomal protein L6, partial	0.82	0.15
Unigene8941	ribosomal protein L4, partial	0.82	0.09
CL1672.Contig2	60S ribosomal protein L10-like	0.73	0.14
CL4437.Contig3	splicing factor, proline- and glutamine-rich	0.62	0.05
CL5572.Contig1	THO complex subunit 4	0.64	0.25
Unigene22920	small nuclear ribonucleoprotein-associated proteins B and B′	0.81	0.12
Unigene15894	malectin	0.75	0.08
**DNA Replication and Repair**			
CL1035.Contig9	histone H3.3	0.66	0.29
Unigene5846	histone H1-β, late embryonic	0.61	0.05
CL5357.Contig1	legumain-like	0.67	0.20
CL64.Contig2	poly(ADP-ribose) polymerase pme-5-like	0.77	0.08
Unigene9968	ATP-binding cassette, sub-family C, member 9-like	0.69	0.01
**Amino Acid Transport and Mechanism**			
CL4631.Contig1	choline dehydrogenase, mitochondrial-like	0.65	0.17
Unigene11760	branched-chain-amino-acid aminotransferase, cytosolic	0.71	0.08
CL3226.Contig1	tyrosine aminotransferase-like	0.66	0.28
Unigene25781	aminopeptidase *N*-like	0.72	0.13
CL12737.Contig1	glutamyl aminopeptidase	0.79	0.16
CL2682.Contig1	cytosolic serine hydroxymethyltransferase	0.73	0.08
CL9582.Contig2	xaa-Pro aminopeptidase 1 isoform X3	0.76	0.09
Unigene36365	betaine homocysteine *S*-methyltransferase 1	0.58	0.22
Unigene3911	betaine homocysteine *S*-methyltransferase 1-like	0.38	0.10
**Lipid Transport and Mechanism**			
Unigene27722	peroxisomal bifunctional enzyme-like	0.78	0.14
Unigene10407	peroxisomal bifunctional enzyme	0.81	0.07
CL8765.Contig2	dihydropteridine reductase	0.74	0.09
CL1598.Contig1	γ-butyrobetaine dioxygenase-like	0.60	0.22
**Carbohydrate Transport and Metabolism**			
Unigene27857	α-mannosidase 2C1-like	0.47	0.24
Unigene18547	pyruvate carboxylase, mitochondrial	0.73	0.15
**Hormonal and Nerve Regulation**			
Unigene25501	thyroid hormone-induced protein B-like	0.41	0.28
CL9528.Contig2	proactivator polypeptide	0.72	0.23
Unigene19435	angiotensin-converting enzyme	0.38	0.18
CL7652.Contig1	potassium channel tetramerization domain containing 6-like	0.37	0.29
CL5238.Contig2	glutamate receptor 1-like	0.68	0.08
**Others/Uncharacterized**			
Unigene1888b1	sterigmatocystin biosynthesis dehydrogenase stcV	0.65	0.26
CL5732.Contig1	*N*-acetylated-α-linked acidic dipeptidase 2-like isoform 1	0.58	0.11
CL9717.Contig2	peroxiredoxin-4-like	0.53	0.07
Unigene25753	homogentisate 1,2-dioxygenase	0.56	0.32
CL2303.Contig2	cathepsin L	0.70	0.26
Unigene8231	α-parvin-like	0.77	0.11
CL7154.Contig1	sorcin	0.82	0.10
Unigene32921	thiopurine *S*-methyltransferase isoform X2	0.75	0.07
CL2660.Contig13	phosphatidylinositol-binding clathrin assembly protein unc-11-like isoform 6	0.80	0.09
Unigene15838	cytochrome P450 2N2	0.70	0.10
CL2540.Contig2	cytochrome P450 2U1-like	0.58	0.05
Unigene23131	oocyst wall protein 4 precursor	0.58	0.10
CL4607.Contig1	uterine-ovary specific-44 protein	0.79	0.14
CL10965.Contig2	MAM and LDL-receptor class A domain-containing protein 2-like	0.73	0.13
Unigene11852	uncharacterized	0.74	0.17
Unigene8201	uncharacterized	0.62	0.21
Unigene63234	uncharacterized	0.55	0.34
Unigene11761	uncharacterized	0.77	0.08
CL11132.Contig3	uncharacterized	0.59	0.23
Unigene23211	uncharacterized	0.77	0.20
CL12015.Contig1	uncharacterized	0.58	0.19
CL709.Contig5	uncharacterized	0.37	0.27
Unigene8605	uncharacterized	0.78	0.03
CL3869.Contig4	uncharacterized	0.58	0.12

### 2.2. Gene Ontology (GO) and Kyoto Encyclopedia of Genes and Genomes (KEGG) Pathway Enrichment Analyses

**Figure 2:**
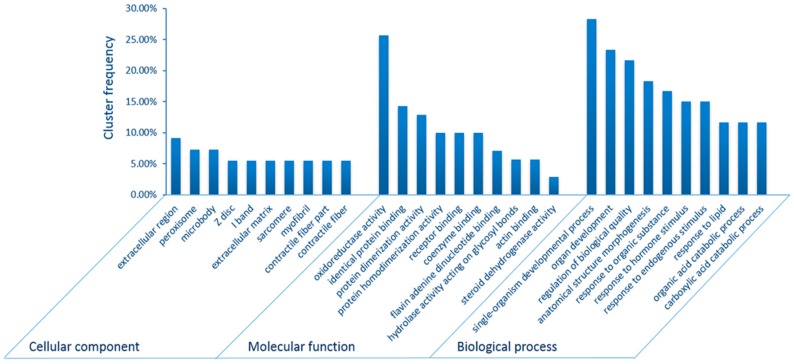
Enriched Gene Ontology (GO) analysis of differentially expressed proteins in the intestine under heat stress. The top 10 enriched GO-terms by cluster frequency in “Cellular component”, “Molecular function” and “Biological process” were shown, respectively.

A GO analysis was performed to evaluate the functions of the differentially expressed proteins. Totals of 15, 12, and 59 categories were enriched in cellular component (CC), molecular function (MF) and biological process (BP) categories, respectively (Table S2). Fiber components (sarcomere, myofibril, contractile fiber part, and contractile fiber), binding functions (identical protein binding, receptor binding, coenzyme binding, and flavin adenine dinucleotide binding, and actin binding) and response processes (response to organic substance, response to hormone stimulus, response to endogenous stimulus, and response to lipid) were of the top 10 enriched GO-terms by cluster frequency in CC, MF and BP, respectively (Figure 2).

A KEGG pathway enrichment analysis revealed seven upregulated pathways, of which xenobiotics metabolism and fatty acid related metabolisms were included (Table 4). Systemic lupus erythematosus and the renin-angiotensin system were identified as downregulated pathways.

**Table 4:** Enriched pathways of different expressed proteins (heat stress (HS) *vs.* Control).

Pathway ID	Pathway Term	All Proteins with Pathway Annotation (2664)	Differential Proteins with Pathway Annotation (100)	*p*-Value
**Upregulated Pathways**				
ko00480	Glutathione metabolism	30 (1.13%)	6 (6%)	1.37 × 10^−3^
ko00980	Metabolism of xenobiotics by cytochrome P450	46 (1.73%)	7 (7%)	1.51 × 10^−3^
ko00071	Fatty acid metabolism	60 (2.25%)	8 (8%)	2.31 × 10^−3^
ko03320	Peroxisome proliferator-activated receptors (PPAR) signaling pathway	64 (2.4%)	8 (8%)	4.35 × 10^−3^
ko00120	Primary bile acid biosynthesis	56 (2.1%)	7 (7%)	8.82 × 10^−3^
ko00982	Drug metabolism-cytochrome P450	51 (1.91%)	6 (6%)	1.10 × 10^−2^
ko05215	Prostate cancer	37 (1.39%)	5 (5%)	1.13 × 10^−2^
**Downregulated Pathways**				
ko05322	Systemic lupus erythematosus	23 (0.86%)	4 (4%)	9.55 × 10^−3^
ko04614	Renin-angiotensin system	51 (1.91%)	6 (6%)	1.10 × 10^−2^

## 3. Discussion

The sea cucumber *A. japonicus* in the northern China experienced the highest temperature between 26 and 30 °C in the field [11]. Besides, *A. japonicus* enters a state of aestivation when the ambient temperature is maintained at 26 °C [3]. Previous reports showed that catalase (CAT) and superoxide dimutase (SOD) activities and HSPs levels varied significantly at 26 °C, indicating that this temperature is beyond the normal temperature limit for *A. japonicus* [3,4,5]. Therefore, we investigate the global protein expression profile under 26 °C heat stress.

### 3.1. Tissue Protection and Detoxification

HSP families play crucial roles protecting organisms against stress by re-establishing normal protein conformation and cellular homeostasis [12]. In our study, heat shock protein 90 (HSP90), HSP70, HSP100, and HSP10 were upregulated 6.10-, 1.56-, 1.41-, and 1.20-fold, respectively (Table 2). The protein family HSP90 helps in the processes of protein folding, degradation and transport, and is involved in cell-signal and cell-cycle control [13,14]. Our previous *A. japonicus* study showed that HSP90 also responds to HS at the mRNA level [5]. HSP70 helps prevent protein aggregation, assists in re-folding of abnormal proteins, and is essential for protein import and translocation processes [15,16]. HSP70 expression increased under heat stress in this study, which agreed with our previous western blot research of HSP70 [17]. HSP100 expression was also upregulated under heat stress. It is now clear that HSP100 plays a major role in thermo-tolerance, particularly in plants [18]. Recent HSP100 studies have focused on its cooperation with HSP70 during protein disaggregation [19]. HSP10 participates in various processes with HSP60, including the stress response and tumorigenesis [20,21]. In our study, HSP10 was more abundantly expressed under heat stress, which also agreed with our previous HSP10 mRNA study [4]. Taken together, our proteomics data show that four HSPs responded significantly to heat stress, indicating that these HSPs played crucial roles in alleviating heat stress in the sea cucumber *A. japonicus*.

Glutathione (GSH) is involved in many biological processes either as a co-factor of enzymatic reactions or as the major thiol-disulfide redox buffer [22]. Furthermore, GSH and GSH-associated metabolism provide important defense from many forms of stress [23]. In our study, phospholipid hydroperoxide peroxidase (GPx4) and three glutathione transferases (GSTs) were upregulated after heat stress in *A. japonicus* (Table 2). GPx4, a 20–22 kDa monomer, reduces hydroperoxides of complex lipids by transferring GSH to glutathione disulfide [22]. This process is crucial for scavenging or reducing excess quantities of reactive oxygen species (ROS), thereby maintaining cell redox homeostasis [23]. GSTs are essential enzymes in GSH metabolism, as GSH forms conjugates with a variety of electrophilic compounds, including various xenobiotic compounds, through the actions of GSTs [24]. The GSH conjugates are then exported out of the cell, which is an important component of detoxification [23]. Therefore, upregulation of GSH metabolic enzymes under heat stress is widely regarded as an essential way that cells protect against toxic damage [22,25,26].

### 3.2. Lipid, Amino Acid and Carbohydrate Metabolism

Fifteen proteins involved in lipid transport and mechanisms were upregulated under heat stress in *A. japonicus*, such as long-chain specific acyl-CoA dehydrogenases (ACADs) and enoyl-CoA hydratases (ECHs). ACADs are a class of enzymes that function to catalyze the initial step of fatty acid β-oxidation in the mitochondria [27]. Long-chain specific ACADs catalyze the breaking of long chain fatty acids into acetyl-CoA molecules. Two long-chain specific ACADs were highly expressed under heat stress in *A. japonicus*, reflecting the increasing demand for fatty acid metabolism. ECHs catalyze the second step of β-oxidation to breakdown fatty acids to produce acetyl-CoA and energy in the form of NADH [28]. These enzymes are highly efficient, allowing cells to metabolize fatty acids into energy very quickly. In our study, 17-β-ECH type 4 and 11-β-ECH were upregulated in the intestinal tissues of *A. japonicus* exposed to heat stress, suggesting a shift to lipid metabolism during energy production. These findings correlate well with previous reports suggesting that upregulation of fatty acid metabolism is an important energy budget strategy in a disadvantageous environment [26,29,30,31].

Nine proteins with roles in amino acid metabolism were less abundant in the HS group (Table 3). Notably, two types of betaine homocysteine *S*-methyltransferase (BHMT), BHMT1 and BHMT1-like, were downregulated 0.58- and 0.38-fold, respectively. BHMTs use betaine to catalyze the conversion of homocysteine (Hcy) to methionine (Met), which helps regulate Hcy levels and Met biosynthesis [32,33]. In our study, the expression of BHMTs decreased significantly under heat stress, which would result in changes in the concentrations of many metabolites and enzymes activities involved in Met, Hcy, and one-carbon metabolism [34]. Together with the reduction in other enzymes involved with amino acid metabolism, we suggest that heat stress may disrupt amino acid homeostasis. Hence, more attention should be paid to the nutrient requirements and amino acid deficiency diseases in sea cucumbers under stress.

In contrast, fewer proteins of carbohydrate transport and metabolism were involved under stress, as only one protein (lactase) was upregulated and two (α-mannosidase 2C1-like and pyruvate carboxylase) were downregulated. Expression of the key enzymes in the glycolytic pathway did not change, such as 6-phosphofructo-2-kinase and pyruvate kinase, indicating that short-term heat stress has no significant influence on this process [26] (Table S1). Pyruvate carboxylase, which synthesizes phosphoenolpyruvate from pyruvate during gluconeogenesis, was downregulated [35]. Thus, gluconeogenesis was likely depressed under heat stress.

### 3.3. Energy Production and Usage

Eight proteins involved in energy production and conversion were upregulated. Electron transfer flavoprotein (ETF) is located on the matrix face of the inner mitochondrial membrane and is a specific electron acceptor [36]. This protein is an important part of the electron transport chain, as it creates an electrochemical proton gradient that drives ATP synthesis. ETF was more abundantly expressed in *A. japonicus* under heat stress, suggesting that regulating HS demands additional energy [25]. However, the electron transport chain is the major site of ROS production, which may be the reason why high temperature increases the ROS levels in the cell [37,38].

Isocitrate dehydrogenase, a critical tricarboxylic acid (TCA) cycle enzyme, catalyzes the oxidative decarboxylation of isocitrate to produce α-ketoglutarate, using NAD+ or NADP+ as a co-factor [39]. Upregulation of isocitrate dehydrogenase accelerates the TCA cycle, suggesting an urgent need for energy. Additionally, isocitrate dehydrogenase is involved in controlling the mitochondrial redox balance and cellular defense against ROS, and overexpression of this enzyme results in protection from ROS-induced damage in mouse cells [40]. Isocitrate dehydrogenase increased in abundance in blue mussel *Mytilus trossulus* under heat stress while decreased in sea urchin *Strongylocentrotus purpuratus* exposed to stressful ultraviolet radiation [41,42]. Hence species may have different approaches to sense and deal with ROS [42].

These findings suggest that high temperature induces increase in the ROS production, which functions as a signal for activating a shift in metabolic pathways to enhance ROS-scavenging in *A. japonicus*.

### 3.4. Transcription and Translation

Our proteomics data show that the majority of proteins involved in transcription and translation were downregulated, including splicing factor, THO complex subunit 4, small nuclear ribonucleoprotein-associated proteins B and B′ and 60S ribosomal proteins. Global transcription and translation decrease in response to most types of cellular stress [29,43,44]. It is estimated that up to 50% of cellular energy, depending on the organism, is consumed in the translation process [45,46]. Hence, this decrease allows for a notable cellular energy savings. Furthermore, reducing protein synthesis avoids exposing nascent polypeptides to denaturing conditions that could further intensify the cellular stress response [44].

Only a few specific proteins participating in protein synthesis were more abundantly expressed in the HS group, such as elongation factor thermo unstable (EF-Tu). Actually, the functions of EF-Tu are not limited to a translation elongation factor but include chaperoning [47]. EF-Tu is an important HS response protein in many species, and high EF-Tu expression is correlated with thermo-tolerance [48,49,50].

### 3.5. Cell Apoptosis and Proliferation

Cell apoptosis signal occurs under heat stress. Apoptosis-inducing factor 1, a ubiquitous mitochondrial flavoprotein that participates in the degradation phase of apoptosis, rose 1.28-fold under heat stress in *A. japonicus* [51]. This result indicate that apoptosis is more predominant under heat stress, which agrees with the apoptotic signals detected in our previous ultrastructural observations, such as condensed chromatin and disappearing cytoplasm [17].

The evidence for decreased cell proliferation is quite clear. For example, two key antiproliferative proteins (prohibitin and autocrine proliferation repressor protein) were upregulated 1.19- and 3.57-fold, respectively [52,53]. Moreover, two types of histone proteins (histone H1 and histone H3.3) are less abundant under heat stress. Histone proteins are responsible for regulating DNA-templating processes, including DNA replication and repair [54]. Therefore, downregulation of histone proteins reflects decreased cell proliferation under stress.

### 3.6. Other Processes

Many other proteins were involved in the *A. japonicus* heat stress response. For example, we identified nine cytoskeletal proteins with decreased expression, suggesting the induction of apoptosis and depressed cell proliferation under heat stress [55,56]. Furthermore, cytoskeletal elements are composed of sarcomeres and reducing their expression decreases muscle contraction under heat stress [57,58]. The levels of hormonal and nerve regulation change under heat stress, which influence metabolism, signal transport, and other physiological functions [59,60,61]. Additionally, many uncharacterized proteins and proteins whose roles in the HS response remain unknown were detected. These results show the complexity of the HS response.

## 4. Materials and Methods

### 4.1. Animals and Samples

*A. japonicus* (mean weight, 99 ± 13 g) were supplied by a commercial farm in Qingdao (Shandong, China) in April 2015. Seawater temperature of the farm was about 13 °C. The sea cucumbers were transported to our laboratory and maintained in seawater tanks (30‰ salinity, 15 °C) for 2 weeks. The sea cucumbers were fed with a formulated diet (5.04% ± 0.19% (*w*/*w*) crude protein, 0.26% ± 0.05% (*w*/*w*) fat, and 72.20% ± 0.19% (*w*/*w*) ash) during the acclimation and experimental periods, and remaining feed was removed daily.

A rapid temperature-change regime was carried out in the treatment tank, using a 2-kW heating rod. The rate of heating was about 2 °C/h. The moment when water temperature rose to 26 °C was regarded as the initial time, and water temperature maintained at 26 °C in the subsequent experiment. Intestinal tissues of *A. japonicus* after a 48 h exposure were sampled as the heat stress (HS) group while those from an untreated tank were sampled as the control (C) group. No sea cucumbers died during the experiment. The intestinal tissues were frozen in liquid nitrogen and stored at −80 °C.

### 4.2. Protein Extraction, Digestion, and iTRAQ Labeling

Three biological replicates of the frozen intestinal tissues were prepared for the iTRAQ analysis. The tissue was ground to powder in liquid nitrogen and dissolved in lysis buffer (7 M urea, 2 M thiourea, 4% CHAPS, and 40 mM Tris-HCl, pH 8.5) containing 1 mM PMSF and 2 mM EDTA. 10 mM DTT was added to the lysis buffer after 5 min. An ultrasound on ice for 15 min was carried out to mix the suspension, which was then centrifuged at 25,000× *g* for 20 min at 4°C. The supernatant was transferred to chilled acetone and precipitated at −20 °C overnight. The supernatant was discarded after centrifugation at 25,000× *g* for 30 min at 4 °C, and the precipitate was washed three times with chilled acetone for 30 min each at 4 °C. The pellets were air-dried and dissolved in lysis buffer using ultrasound. The supernatant was reduced with 10 mM DTT at 56 °C for 1 h after centrifugation at 25,000× *g* for 30 min at 4 °C and alkylated immediately with 55 mM iodoacetamide in the dark at room temperature for 1 h. The treated proteins were precipitated in acetone at −20 °C for 3 h. The proteins were dissolved in buffer containing 1 mM PMSF and 2 mM EDTA using ultrasound after centrifugation at 25,000× g for 20 min at 4 °C and air-drying. The proteins were recovered after centrifugation at 25,000× g for 20 min at 4 °C and quantified using the Bradford method.

The protein samples were digested with Trypsin Gold (Promega, Madison, WI, USA) at 37 °C for 16 h, and the peptides were dried by vacuum centrifugation. An isobaric tag was labeled to the control (113, 114 and 116 Da) and HS samples (118, 119 and 121 Da), following the manufacturer’s instructions for the iTRAQ 8-plex reagents (Applied Biosystems, Foster City, CA, USA).

### 4.3. Fractionation by Strong Cation Exchange Chromatography (SCX) and Liquid Chromatography-Tandem Mass Spectrometry (LC-MS/MS) Analysis

The labeled samples were fractionated on a SCX column using the LC-20AB high performance liquid chromatography (HPLC) pump system (Shimadzu, Kyoto, Japan). The peptides were eluted with a gradient of buffer A (25 mM NaH_2_PO_4_ in 25% ACN, pH 2.7) and buffer B (25 mM NaH_2_PO_4_ and 1 M KCl in 25% ACN, pH 2.7). The specific fractionating procedures were as follows: 100% buffer A for the first 10 min, 5%–60% buffer B for 27 min, 60%–100% buffer B for 1 min, and 100% buffer B for 1 min. Absorbance of the eluate was measured at 214 nm, and fractions were collected every min. The eluted peptides were desalted with a Strata X C18 column (Phenomenex, Torrance, CA, USA) and vacuum-dried.

A LC-20AD nanoHPLC (Shimadzu, Kyoto, Japan) and a 10 cm eluting C18 column were used to analyze the peptide fractions. Mass spectrometry data were acquired with the Triple TOF 5600 system (AB SCIEX, Concord, ON, Canada) fitted with the Nanospray III source (AB SCIEX) and a pulled quartz tip emitter (New Objectives, Woburn, MA, USA).

### 4.4. Protein Identification and Quantification

The raw LC-MS/MS data were converted to MGF files using Proteome Discovery 1.2 (Thermo, Pittsburgh, PA, USA). The proteins were identified using Mascot search engine 2.3.02 (Matrix Science, London, UK) with the *A. japonicus* transcriptomics database containing 30,622 sequences. Proteins containing at least two unique spectra were used for the follow-up quantification analysis. The quantitative protein ratios were weighted and normalized in Mascot. We only identified proteins with *p*-values <0.05 and fold changes >1.20 or <0.83 as being differentially expressed [62].

### 4.5. GO and KEGG Pathway Enrichment Analyses

The GO and KEGG databases were used to classify and group the identified proteins [63,64]. The hypergeometric test was used to identify significantly enriched GO terms and pathways of differentially expressed proteins. A *p*-value <0.05 was considered as significant.

## 5. Conclusions

This study provides a global view of the proteins differentially expressed in the intestinal tissues of *A. japonicus* under heat stress using the iTRAQ technique. Heat stress influences the expression of proteins involved in various biological processes, such as tissue protection and detoxification, lipid and amino acid metabolism, energy production and usage, transcription and translation, cell apoptosis, and cell proliferation. These results reveal possible molecular events in *A. japonicus* under heat stress.

## Supplementary Materials

Supplementary materials can be found at http://www.mdpi.com/1422-0067/17/2/150/s1.
