# Supplementary material for: Understanding the Heat Shock Response in the Sea Cucumber Apostichopus japonicus, Using iTRAQ-Based Proteomics
Source: Int J Mol Sci. 2016 Feb 4;17(2):150. doi: 10.3390/ijms17020150 (PMC4783884; doi:10.3390/ijms17020150)
Supplement: Supplementary file 1 [file ijms-17-00150-s001.zip › ijms-109999-Supplementary Materials/TableS2 Enriched GO analysis.pdf]

Supplementary Materials: Understanding the Heat Shock Response in the Sea Cucumber *Apostichopus Japonicus*, Using iTRAQ-Based Proteomics

Dongxue Xu, Lina Sun, Shilin Liu, Libin Zhang and Hongsheng Yang

Table S2. Go enriched analysis of different expressed proteins (HS *vs.* Control).

Gene Ontology Terms	Reference (N/M)	Cluster (n/m)	*p*-Value	Gene Ontology Terms	Reference (N/M)	Cluster (n/m)	*p*-Value
**Cellular Component (15 Categories)**				**Biology Progress (59 Categories) (Continued)**			
Z disc	12/1590, 0.8%	3/55, 5.5%	6.91×10^{-3}	organ development	210/1793, 11.7%	14/60, 23.3%	7.39×10^{-3}
I band	14/1590, 0.9%	3/55, 5.5%	1.09×10^{-2}	response to estrogen stimulus	14/1793, 0.8%	3/60, 5.0%	9.97×10^{-3}
postsynaptic membrane	6/1590,0.4%	2/55,, 3.6%	1.61×10^{-2}	reproductive structure development	14/1793, 0.8%	3/60, 5.0%	9.97×10^{-3}
extracellular region	46/1590,2.9%	5/55, 9.1%	1.91×10^{-2}	reproductive system development	14/1793, 0.8%	3/60, 5.0%	9.97×10^{-3}
brush border	7/1590,0.4%	2/55, 3.6%	2.21×10^{-2}	L-phenylalanine metabolic process	5/1793, 0.3%	2/60, 3.3%	1.03×10^{-2}
extracellular matrix	18/1590, 1.1%	3/55, 5.5%	2.21×10^{-2}	L-phenylalanine catabolic process	5/1793, 0.3%	2/60, 3.3%	1.03×10^{-2}
sarcomere	21/1590, 1.3%	3/55, 5.5%	3.35×10^{-2}	regulation of striated muscle contraction	5/1793, 0.3%	2/60, 3.3%	1.03×10^{-2}
extracellular matrix part	9/1590, 0.6%	2/55, 3.6%	3.62×10^{-2}	intracellular steroid hormone receptor signaling pathway	5/1793, 0.3%	2/60, 3.3%	1.03×10^{-2}
synaptic membrane	9/1590, 0.6%	2/55, 3.6%	3.62×10^{-2}	peptide catabolic process	5/1793, 0.3%	2/60, 3.3%	1.03×10^{-2}
myofibril	22/1590, 1.4%	3/55, 5.5%	3.79×10^{-2}	female sex differentiation	5/1793, 0.3%	2/60, 3.3%	1.03×10^{-2}
contractile fiber part	22/1590, 1.4%	3/55, 5.5%	3.79×10^{-2}	organic acid catabolic process	75/1793, 4.2%	7/60, 11.7%	1.10×10^{-2}
peroxisome	39/1590, 2.5%	4/55, 7.3%	4.29×10^{-2}	carboxylic acid catabolic process	75/1793, 4.2%	7/60, 11.7%	1.10×10^{-2}
microbody	39/1590, 2.5%	4/55, 7.3%	4.29×10^{-2}	sex differentiation	15/1793, 0.8%	3/60, 5.0%	1.22×10^{-2}
proteinaceous extracellular matrix	10/1590, 0.6%	2/55, 3.6%	4.43×10^{-2}	kidney development	6/1793, 0.3%	2/60, 3.3%	1.51×10^{-2}
contractile fiber	24/1590, 1.5%	3/55, 5.5%	4.74×10^{-2}	cellular iron ion homeostasis	6/1793, 0.3%	2/60, 3.3%	1.51×10^{-2}
**Molecular Function (12 Categories)**				regulation of muscle contraction	6/1793, 0.3%	2/60, 3.3%	1.51×10^{-2}
identical protein binding	92/1888, 4.9%	10/70, 14.3%	1.62×10^{-3}	response to organic cyclic compound	48/1793, 2.7%	5/60, 8.3%	2.01×10^{-2}
protein dimerization activity	90/1888, 4.8%	9/70, 12.9%	4.99×10^{-3}	regulation of system process	32/1793, 1.8%	4/60, 6.7%	2.02×10^{-2}
protein homodimerization activity	62/1888, 3.3%	7/70, 10.0%	6.87×10^{-3}	regulation of receptor-mediated endocytosis	7/1793, 0.4%	2/60, 3.3%	2.08×10^{-2}

Table S2. *Cont.*

Gene Ontology Terms	Reference (N/M)	Cluster (n/m)	*p*-Value	Gene Ontology Terms	Reference (N/M)	Cluster (n/m)	*p*-Value
steroid dehydrogenase activity	4/1888, 0.2%	2/70, 2.9%	7.75×10^{-3}	small molecule catabolic process	87/1793, 4.9%	7/60, 11.7%	2.36×10^{-2}
oxidoreductase activity	275/1888, 14.6%	18/70, 25.7%	8.87×10^{-3}	single-organism catabolic process	87/1793, 4.9%	7/60, 11.7%	2.36×10^{-2}
flavin adenine dinucleotide binding	36/1888, 1.9%	5/70, 7.1%	9.34×10^{-3}	cellular metal ion homeostasis	19/1793, 1.1%	3/60, 5.0%	2.36×10^{-2}
3-hydroxyacyl-CoA dehydrogenase activity	6/1888, 0.3%	2/70, 2.9%	1.85×10^{-2}	metal ion homeostasis	19/1793, 1.1%	3/60, 5.0%	2.36×10^{-2}
peroxiredoxin activity	6/1888, 0.3%	2/70, 2.9%	1.85×10^{-2}	regulation of biological quality	220/1793, 12.3%	13/60, 21.7%	2.60×10^{-2}
receptor binding	76/1888, 4.0%	7/70, 10.0%	2.02×10^{-2}	hormone metabolic process	20/1793, 1.1%	3/60, 5.0%	2.71×10^{-2}
hydrolase activity, acting on glycosyl bonds	33/1888, 1.7%	4/70, 5.7%	3.15×10^{-2}	sarcomere organization	8/1793, 0.4%	2/60, 3.3%	2.71×10^{-2}
coenzyme binding	86/1888, 4.6%	7/70, 10.0%	3.70×10^{-2}	renal system development	8/1793, 0.4%	2/60, 3.3%	2.71×10^{-2}
actin binding	36/1888, 1.9%	4/70, 5.7%	4.17×10^{-2}	single-organism developmental process	319/1793, 17.8%	17/60, 28.3%	2.74×10^{-2}
**Biology Progress (59 Categories)**				response to organic substance	158/1793, 8.8%	10/60, 16.7%	3.34×10^{-2}
response to hormone stimulus	64/1793, 3.6%	9/60, 15.0%	1.86×10^{-4}	regulation of muscle system process	9/1793, 0.5%	2/60, 3.3%	3.41×10^{-2}
response to metal ion	34/1793, 1.9%	6/60, 10.0%	7.06×10^{-4}	peptide metabolic process	22/1793, 1.2%	3/60, 5.0%	3.49×10^{-2}
response to lipid	48/1793, 2.7%	7/60, 11.7%	8.27×10^{-4}	cellular amino acid catabolic process	38/1793, 2.1%	4/60, 6.7%	3.58×10^{-2}
aromatic amino acid family catabolic process	8/1793, 0.4%	3/60, 5.0%	1.77×10^{-3}	regulation of growth	38/1793, 2.1%	4/60, 6.7%	3.58×10^{-2}
gonad development	9/1793, 0.5%	3/60, 5.0%	2.59×10^{-3}	muscle structure development	39/1793, 2.2%	4/60, 6.7%	3.89×10^{-2}
response to lead ion	3/1793, 0.2%	2/60, 3.3%	3.23×10^{-3}	2-oxoglutarate metabolic process	10/1793, 0.6%	2/60, 3.3%	4.17×10^{-2}
response to endogenous stimulus	95/1793, 5.3%	9/60, 15.0%	3.50×10^{-3}	regulation of heart contraction	10/1793, 0.6%	2/60, 3.3%	4.17×10^{-2}
response to steroid hormone stimulus	32/1793, 1.8%	5/60, 8.3%	3.56×10^{-3}	response to temperature stimulus	10/1793, 0.6%	2/60, 3.3%	4.17×10^{-2}
aromatic amino acid family metabolic process	10/1793, 0.6%	3/60, 5.0%	3.62×10^{-3}	regulation of hormone levels	24/1793, 1.3%	3/60, 5.0%	4.38×10^{-2}
development of primary sexual characteristics	10/1793, 0.6%	3/60, 5.0%	3.62×10^{-3}	cellular cation homeostasis	24/1793, 1.3%	3/60, 5.0%	4.38×10^{-2}
tyrosine metabolic process	4/1793, 0.2%	2/60, 3.3%	6.33×10^{-3}	anatomical structure morphogenesis	190/1793, 10.6%	11/60, 18.3%	4.60×10^{-2}
female gonad development	4/1793, 0.2%	2/60, 3.3%	6.33×10^{-3}	response to abiotic stimulus	80/1793, 4.5%	6/60, 10.0%	4.77×10^{-2}
response to aluminum ion	4/1793, 0.2%	2/60, 3.3%	6.33×10^{-3}	urogenital system development	11/1793, 0.6%	2/60, 3.3%	4.99×10^{-2}
development of primary female sexual characteristics	4/1793, 0.2%	2/60, 3.3%	6.33×10^{-3}	amino-acid betaine metabolic process	11/1793, 0.6%	2/60, 3.3%	4.99×10^{-2}
response to inorganic substance	52/1793, 2.9%	6/60, 10.0%	6.68×10^{-3}	iron ion homeostasis	11/1793, 0.6%	2/60, 3.3%	4.99×10^{-2}

M is the number of all proteins with GO annotation; m is the number of differential expressed proteins in M; N is the number of all proteins that are annotated to the certain GO terms; n is the number of differential expressed proteins in N.
